# Speed-Breeding System in Soybean: Integrating Off-Site Generation Advancement, Fresh Seeding, and Marker-Assisted Selection

**DOI:** 10.3389/fpls.2021.717077

**Published:** 2021-08-17

**Authors:** Yudong Fang, Liwei Wang, Enoch Sapey, Shuai Fu, Tingting Wu, Haiyan Zeng, Xuegang Sun, Shuqing Qian, Mohammad Abdul Awal Khan, Shan Yuan, Cunxiang Wu, Wensheng Hou, Shi Sun, Tianfu Han

**Affiliations:** ^1^MARA Key Laboratory of Soybean Biology (Beijing), Institute of Crop Sciences, Chinese Academy of Agricultural Sciences, Beijing, China; ^2^MOA Key Laboratory of Soybean Biology, Institute of Crop Sciences, Chinese Academy of Agricultural Sciences, Beijing, China

**Keywords:** generation advancement, off-site nursery, fresh seeding method, marker-assisted selection, speed breeding system

## Abstract

Speed breeding by artificial control of photothermal conditions facilitates generation advancement but was limited in scale and cost. In this study, we demonstrated a cost-saving off-site summer nursery pattern, taking full advantage of shorter daylength and higher temperature with lower latitude compared to the origin of the soybean cultivars used in the study. This substantially reduced the generation cycles under totally natural conditions. Using this approach, two generations of soybean cultivars from Northeastern Spring Planting Region (NE) and Yellow-Huai-Hai Valleys Summer Planting Region (YHH) were successfully obtained in Beijing and Hainan, respectively, compared to one generation in origin. Fresh-seeding method was also used to further shorten the generation duration by 7–10 days, thereby allowing at least four generations per year. Using DNA markers to define haplotypes of maturity genes *E1–E4*, we proposed a model to predict the optimum adaptation region of the advanced generation lines. Taken together, we present a speed-breeding methodology combining off-site nursery, fresh-seeding method, and marker-assisted selection, aimed at accelerating soybean improvement.

## Introduction

Soybean [*Glycine max* (L.) Merrill] is one of the important oil crops in China due to its industrial use and domestic consumption. There is an urgent demand for high-yielding soybean cultivars to reduce the importation from other countries. In developing superior cultivars, cross-breeding is an effective breeding method in a great majority of crops, including soybean, and usually more productive and/or with other desirable characteristics from rich genetic variation by hybridization (Gai et al., 2015). Nonetheless, cross-breeding in soybean is time-consuming, generally requires at least eight generations from the crossing of selected parents to genetically stable lines for selection and evaluation. More than four generations are required to develop pure lines in this process. This slow development rate is attributed partially to the lengthy generation cycles, which seriously retards the improvement in soybean yield, quality, and resistance to pest and diseases (Forster et al., 2014; Shuai et al., 2017; Fang and Han, 2019). Hence, shortening the breeding cycle through accelerating generation turnover is an issue of major importance in soybean breeding.

Greenhouse strategy allows specific adjustments regarding daylength, temperature, CO_2_ concentration, and other climatic factors to adapt or accelerate plant development and finally shortens the generation time (Wu, 1961; Kothari et al., 2011; De La Fuente et al., 2013; Alahmad et al., 2018; Ghosh et al., 2018; Li et al., 2018; Watson et al., 2018; Jahne et al., 2020). However, with the supplements of equipment and electricity, greenhouse strategy is costly and size limited. Besides, since 1960s, winter nursery in Hainan Island has been widely used, which provides soybean breeders an opportunity to get one more generation during the off-season in north China (from November to April). Even so, two generations per year in Hainan is not enough, hence there still is an urgent need for a more economic and efficient strategy to speed up soybean breeding.

Another attempt to shorten the generation time is the fresh-pod–picking method (Fang and Han, 2019). As the plants reach R6 or full-seed stage, the most-mature pods are picked, and the seeds dried before sowing. In combination with single-seed descent (Chahal and Gosal, 2002), the generation time could be extremely reduced through harvesting small number of immature seeds to sidestep the ripening stage (Chang and Han, 2000).

Ineffective selection could be made directly based on phenotypes during off-season nursery, because phenotypic variation for important agronomic traits (such as growth period, plant height, and yield) among the populations would decrease (Fei et al., 2009). Instead, marker-assisted selection (MAS) could be used as a supplement for the phenotypic selection, while desirable individuals would be selected by genotypes without phenotypes of homozygous lines (Xu and Crouch, 2008). Previous studies showed that haplotype combinations at the *E1–E4* loci can explain more than 60% of variation in flowering time (Liu et al., 2008; Xia, 2013) and that there is a high correlation between *E1* and *E4* genotypes and growth period, and latitudinal adaptability (Jia et al., 2014; Jiang et al., 2014) in soybean. Therefore, *E1–E4* genotype identification of hybrid progenies during speed breeding, instead of phenotype identification, could facilitate the breeding process.

In this study, we developed a flexible protocol called off-site summer nursery to shorten the generation length under natural conditions, using the progenies of cross combinations from different ecotype cultivars as breeding materials. Besides, we found that the fresh-seeding method can further shorten the generation interval in soybean by sidestepping the drying duration of fresh seeds. We also proposed a prediction model of growth period phenotypes in advance with *E1–E4* genotypes after generation advancement and production of genetically stable lines. This significantly improved the selection efficiency. All together, we presented a speed-breeding system combining off-site generation advancement, fresh seeding, and MAS, which greatly reduces generation length and facilitates soybean breeding and research programs at low cost.

## Materials and Methods

### Summer Nursery

Soybean hybrid progenies of diverse ecotypes from Northeastern Spring Planting Region (NE) and Yellow–Huai–Hai Valleys Summer Planting Region (YHH) were used in this study (Supplementary Table 1). Soybean hybrid progenies from NE were generated by crossing of cultivars from Mohe (Heilongjiang province), Ganhe (Inner Mongolia), Suihua (Heilongjiang province), Changchun (Jilin province), and Shenyang (Liaoning province), while soybean hybrid progenies from YHH were generated by crossing of cultivars from Beijing and Xinxiang (Henan province).

**FIGURE 1:**
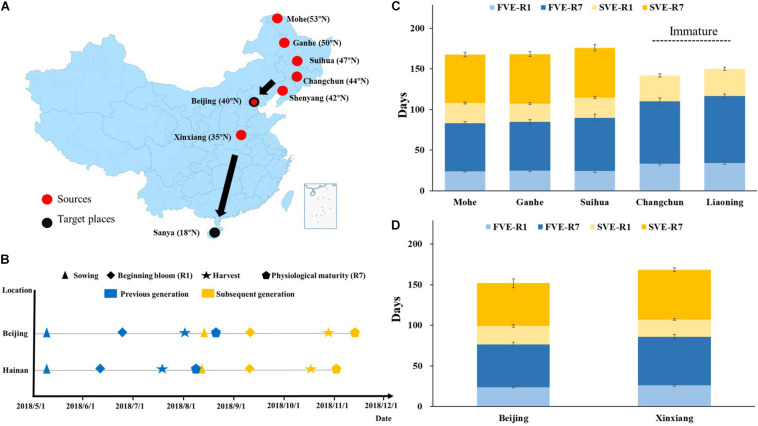
Development of summer nursery in soybean. **(A)** The material sources and planting locations in summer nursery. The progenies of 34 cross combinations from Mohe, Ganhe, Suihua, Changchun, and Shenyang were planted in Beijing. The progenies of eight cross combinations from Beijing and Xinxiang were planted in Hainan. **(B)** Timeline of soybean development during summer nursery in Beijing and Hainan. Blue color symbols represent the developmental stages of the first generation, and the yellow color symbols represent that of the subsequent generation. Developmental stages of summer-sown soybean progenies **(C)** from Northeastern Spring Planting Region or **(D)** from Yellow–Huai–Hai Valleys Summer Planting Region planted in Hainan. F, first generation; S, subsequent generation; VE, emergence; R1, beginning bloom; R7, beginning maturity.

Summer nursery trials were carried out in two parts. Part one: the hybrid progenies from NE were sown on May 9, 2018, in Beipuchang farm of Chinese Academy of Agricultural Sciences (CAAS) in Beijing (39°58′N, 116°20′E). Part two: the hybrid progenies from YHH were sown on May 9, 2018, in Nanbin Farm of CAAS in Sanya, Hainan province (18°27′N, 109°11′E) (Figure 1A).

Fresh pods with the fastest ripening and plumpest seeds on each plant, were harvested after more than 80% of the plants reached the full-seed stage (R6). The pods were dried for 7–10 days in the shade at a temperature of 25–35°C and humidity ≤ 30%. Finally, seeds were detached from dried pods and sown immediately following the same procedure. During the experiment, growth stages were recorded as described by Fehr and Caviness (1977).

### Fresh Seeding Tests

Five soybean varieties, including an early maturing variety Heihe 27, three intermediate maturing varieties Zhonghuang 70, Zhonghuang 325, and Zhonghuang 314, and a late maturing variety Zigongdongdou were used as materials. The varieties were supplied by Ministry of Agriculture and Rural Affairs (MARA) Key Laboratory of Soybean Biology, Institute of Crop Sciences, CAAS.

Seeding was done in Nanbin Farm on November 11, 2018. Harvesting was done by handpicking at six pod developmental stages: (I) Early-seed pod (ES): Pods containing approximately 20% of their maximum weight in fresh seeds at R5.5 stage; (II) Medium-seed pod (MS): Pods containing approximately 40% of their maximum weight in fresh seeds at R5.7 stage; (III) Full-seed pod (FS): Pods fully filled with green seeds at R6 stage; (IV) Green-yellow pod (GYP): Pods with a maximum weight of fresh seeds at R6.5 stage; (V) Yellow pod (YP): Pods with a brownish skin that was not yet round at R7 stage; and (VI) Brown pod (BP): Pods with a brown skin and matured seeds at R8 stage. At each harvest stage, three replicates of 100 seeds were randomly selected to measure the weight and moisture content of seeds and pods before and after oven-drying at 44°C for 96 h. The remaining seeds or harvested pods were dried in two schemes: (1) seeds were carefully extracted from the pods before drying (depodded); (2) seeds were dried inside intact pods (podded). The seeds or pods were left air-dried at an ambient temperature (27 ± 2°C).

Seed germination tests were performed as described by the International Seed Testing Association (1999) protocols with some modifications. Three replicates of 50 seeds were used. The percentage of normal seedlings was determined as the radicle emerged from the seed coat more than 1 mm.

Analysis of variance (ANOVA) was performed by Microsoft Excel 2016. Means were separated by Duncan’s multiple range test at *P* < 0.05, where the *F*-test was significant.

### Genotyping and Phenotyping

A total of 262 hybrid progenies (F_6_) from Mohe were genotyped for *E1–E4* maturity genes. They were also evaluated on the field for growth period traits in two locations.

InDels were detected by fragment-specific PCR and gel electrophoresis, and SNP sites were detected by Kompetitive Allele-Specific PCR (KASP), according to a previously reported protocol (Liu et al., 2020). All primers for sequencing and genotyping are provided in Supplementary Table 2. The PCR amplification products were scanned by FAM/VIC/ROM of BiOTek (SYNERGY/H1 microplate reader), and scanned data were detected by Kluster Caller typing software.

The hybrid progenies (F_6_) were sown on May 9, 2018, in Ganhe Farm, Molidawa Banner, Inner Mongolia (49°27′N, 124°40′E) and Beijicun Village, Mohe City, Heilongjiang province (53°27′N, 122°24′E), respectively, for phenotypic evaluation (growth period). According to descriptions of growth stage in Fehr and Caviness (1977), VE, R1, and R7 were investigated.

## Results

### Off-Site Summer Nursery Shortens the Soybean Breeding Cycle

To explore whether the hybrid progenies from different ecological regions can achieve two generations in summer in lower latitude with shorter day length, hybrid progenies from NE and YHH were planted in Beijing and Sanya under natural conditions, respectively. The daylength and ambient temperature in Beijing and Sanya during soybean summer nursery in 2018 are shown in Supplementary Figure 1.

The hybrid progenies from Mohe, Ganhe, and Suihua were sown on May 9, 2018. The plants reached the beginning bloom stage (R1) at 24.2 ± 1.2, 24.6 ± 1.0, and 24.4 ± 2.2 days after emergence (DAE), respectively (Figure 1C). They began maturity at 83.2 ± 1.9, 84.6 ± 3.1, and 89.8 ± 4.6 DAE, and were harvested by fresh-pod picking on August 1, 2018 (79 DAE). The subsequent generations of these materials were sown on August 8, 2018, and the plants reached R1 at 24.9 ± 1.1, 22.5 ± 1.2, and 25.0 ± 1.3 DAE, respectively. They began maturity at 84.6 ± 2.9, 83.6 ± 3.2, and 86.2 ± 3.8 DAE, respectively, and were harvested by fresh-pod picking on November 1, 2018 (80 DAE). In summary, two generations were accomplished from May 9 to November 1, 2018 (Figure 1B) in hybrid progenies from Mohe, Ganhe, and Suihua located in the north part of northeast China. However, the hybrid progenies from Changchun and Shenyang did not complete two generations in summer of Beijing. None of the second generation of those materials reached beginning maturity stage (R7) and the pod has not developed well before frost (November 16).

The hybrid progenies from YHH were sown on May 9, 2018, in Sanya, reached R1 at 23.9 ± 3.0 and 26.2 ± 2.0 DAE, respectively (Figure 1D). They began maturity at 76.6 ± 5.5 and 85.7 ± 3.3 DAE and were harvested by fresh-pod picking on July 7, 2018 (68 DAE). The next generations of those materials were sown on August 9, 2018, reached R1 at 22.6 ± 1.7 and 21.5 ± 1.1 DAE. They began maturity at 75.2 ± 5.6 and 82.9 ± 2.1 DAE, and were finally harvested by fresh-pod picking on October 10, 2018 (74 DAE). In summary, all of the hybrid progenies from YHH completed two generations from May 9 to October 10, 2018, in Sanya (Figure 1B).

### Fresh Seeding Method Shortens the Soybean Growth Cycle

**FIGURE 2:**
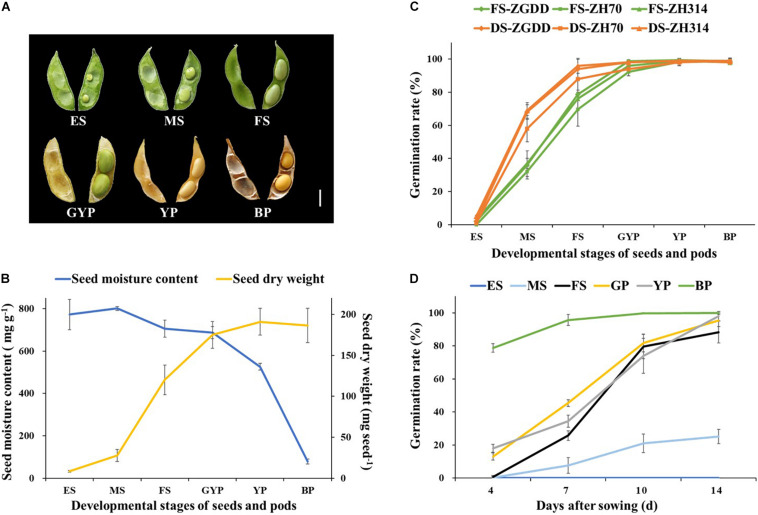
The developmental status and the germination rate of seeds harvested at different reproductive stages. **(A)** Images of pods and seeds harvested at different reproductive stages. **(B)** The moisture content and dry weight of seeds harvested at different reproductive stages. **(C)** The comparison of germination rate between the freshly sown seeds (FS) after shelling and the desiccated seeds (DS). **(D)** The germination rate of the fresh seeds in different days after sowing. The seeds of soybean (*cv*. Heihe27) were sown immediately after shelling. Developmental stages of seeds and pods: ES, early seed pod; MS, medium-seed pod; FS, full-seed pod; GYP, green-yellow pod; YP, yellow pod; BP, brown pod. Scale bar = 1 cm. Values are means ± SD (*n* ≥ 5).

In an attempt to further shorten the generation length of soybean, a fresh-pod–picking method was developed, which could save 1 month in each generation. We first tested the germination of seeds picked from different pod developmental stages (Figure 2A). The dry matter content of FS picked at R6 stage was more than 60%, and then reached the maximum dry weight in YP, as the water content began to drop sharply in GYP (Figure 2B).

As a result, the germination percentage of fresh and dried seeds increased with increasing seed maturity (Figure 2C). The germination rate of dried and fresh seeds harvested at the FS stage reached 88.0 ± 2.6 and 69.7 ± 10.3%, respectively. Furthermore, the difference between the two treatments was not statistically significant, indicating that the fresh seeds harvested at the FS stage have a great germination capacity. There was no significant difference (*P* > 0.05) among different soybean genotypes. As seed maturity progressed to FS, seed germination significantly improved across all the varieties. Therefore, instead of drying seeds after fresh-pod picking, directly sowing fresh seeds harvested at the FS stage could save the drying time, which further advances the sowing time of the next generation by 7–10 days without decreasing the seed germination rate.

To evaluate the germination ability of fresh seeds detached from different developmental pods, we investigated the germination rate on days 4, 7, 10, and 14 after sowing. As shown in Figure 2D, the germination rate of fresh seeds harvested at the BP stage was 78.7 ± 2.6% on the 4th day after sowing (DAS) and reached the maximum on the 10th day. In the meantime, the germination rate of fresh seeds harvested at the GYP and YP stages were only 18.0 ± 2.4 and 13.0 ± 2.2% on the 4th DAS, and reached the maximum, 95.3 ± 3.7 and 98.0 ± 2.8% on the 14th day, respectively. The germination rate of fresh seeds harvested at the FS stage was almost 0 on the 4th DAS but reached the highest value of 88.3 ± 6.5% on the 14th DAS.

**FIGURE 3:**
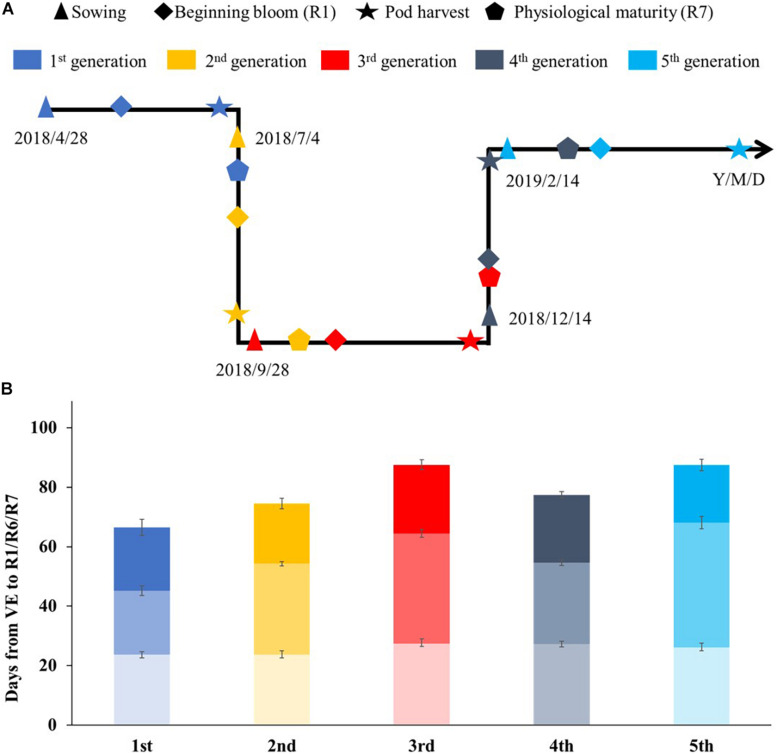
The growth periods of soybean *cv*. Heihe27 under natural conditions (May to October) and glasshouse (November to April) in Beijing, 2018–2019. **(A)** Timeline of soybean development during summer nursery in Beijing and Hainan. **(B)** Developmental stages of Heihe27 planted in Beijing. VE, emergence; R1, beginning bloom; R7, beginning maturity.

We further investigated how many generations can be obtained in 1 year using the fresh-seeding method. Super-early maturity variety Heihe27 was planted in the greenhouse. After recurrent planting, we advanced five generations from April 28, 2018, to May 1, 2019 (Figure 3). The average generation span was about 73 days.

### Marker-Assisted Selection Advances the Soybean Breeding Program

After examining the haplotypes of maturity genes of *E1–E4* and the growth periods of the hybrid progenies (F_6_) in different locations, 211 individual lines were selected for further analysis. We identified three alleles of *E1* (*e1-as*, *e1-fs*, and *e1-ns*), one allele of *E2* (*e2-ns*), three alleles of *E3* (*e3-fs*, *e3-ns*, and *e3-tr*), and four alleles of *E4* (*e4-new*, *e4-kes*, *e4-SORE-1*, and *E4*) in the 211 individual lines for phenotyping. Eighteen haplotypes of *E1–E4* were identified in the hybrid progenies (Supplementary Table 3). Based on the previous studies (Jiang et al., 2014; Li et al., 2017; Liu et al., 2020), we presumed a model to predict the growth period phenotype and maturity groups and divided the hybrid progenies into four groups: (1) super-early group: with the genotypes of *e1-fs/ e2-ns/ e3-tr*, *e3-ns*, *e3-fs/ e4-kes*, *e4-new*, and *e4-SORE-1*; (2) extremely early group: *e1-nl/ e2-ns/ e3-tr*, *e3-ns*, *e3-fs/ e4-kes*, *e4-new*, and *e4-SORE-1*; (3) early group: *e1-as/ e2-ns/ e3-tr*, *e3-ns*, *e3-fs/ e4-kes*, *e4-new*, and *e4-SORE-1*; and (4) mid-early group: *e1-fs*, *e1-nl*, *e1-as/ e2-ns/ e3-tr*, *e3-ns*, and *e3-fs/ E4*.

**FIGURE 4:**
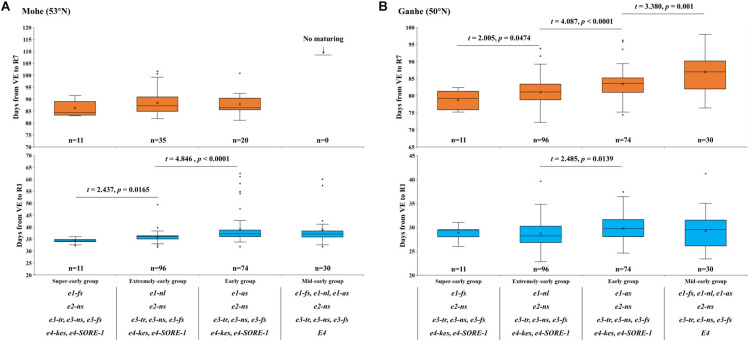
Flowering time (days from VE to R1) and maturity time (days from VE to R7) of different E allele combinations in **(A)** Mohe (53°N) and **(B)** Ganhe (50°N). VE, emergence; R1, beginning bloom; R7, beginning maturity. The *t* value and *P* value for two-tailed *t*-test are shown above the box plot.

The phenotypic characterization of 211 hybrid progenies in Mohe showed that the flowering time of super-early hybrid progenies was significantly (*P* < 0.05) earlier than that of extremely-early hybrid progenies (Figure 4A and Supplementary Figure 2). The flowering time of super-early and extremely early hybrid progenies from the north part of NE was also significantly (*P* < 0.05) earlier than that of the early hybrid progenies from the middle or south part of NE. Except for the super-early hybrid progenies (11/11), the other three groups did not reach maturity normally: 37.2% (35/96) of the predicted extremely early hybrid progenies and 27.0% (20/74) of the predicted early hybrid progenies reached R7, while none (0/30) of the predicted mid-early hybrid progenies hardly mature normally in Mohe. Furthermore, the progression to R7 revealed that the predicted super-early hybrid progenies with the genotypes *e1-fs*, *e2-ns*, *e3-tr/ e3-ns/ e3-fs*, and *e4-kes/ e4-SORE-1* were suitable for cultivation in Beijicun of Mohe, the northernmost village of China, and the higher latitude regions.

The phenotypic characterization of 211 hybrid progenies in Ganhe Farm (Figure 4B) showed that the flowering time of extremely early hybrid progenies were significantly (*P* < 0.05) earlier than that of the early-group hybrid progenies. As expected, all of the predicted super-early hybrid progenies reached R7 in Ganhe, while more than 97.5% of the extremely early and early group hybrid progenies matured normally, showing that predicted extremely early and early group hybrid progenies were suitable for breeding selection in Ganhe. Additionally, the number of mid-early group hybrid progenies (66.7%) progressing to R7 in Ganhe revealed that the predicted mid-early group hybrid progenies were suitable for cultivation in Ganhe or the region further south.

## Discussion

Short daylength and high temperature are the superlative environments for accelerating the growth and development of short-day plants such as soybean (Mao et al., 2017). In China, winter nursery in tropical Hainan Island provided soybean breeders an opportunity to increase one or two additional generations during the off-season in north China (from November to April) (Wu, 1961; Kothari et al., 2011). In this study, we tried to speed up breeding of NE soybean by summer nursery in the YHH regions. As shown in the results, the daylength and temperature in Beijing (Supplementary Figure 1) were ideal for the rapid growth of hybrid progenies of super-early, extremely early, and early group from NE (Figure 1). However, the hybrid progenies classified as mid-early and late groups from NE could not attain two generations in Beijing. Therefore, in order to complete two generations in summer, the hybrids from these groups were recommended to be planted in lower latitude regions with shorter photoperiod and higher accumulated temperature, such as Anhui (29–34°N) or Jiangsu (30–35°N) in China. Our study also revealed that hybrid progenies from YHH can accomplish two generations in Hainan during summer. NE and YHH regions account for more than 75% of the soybean production in China. Soybean varieties in these two regions are within the maturity group (MG) IV, which is the main soybean genotype (Song et al., 2019) cultivated in North America and other mid- and high-latitude regions in the world. Thus, the speed-breeding methodology presented in our study has universal applicability. However, there was one limitation that the germination of the second generation was affected by heavy rains. Therefore, indoor seeding or drainage systems with deep trenches should be available to eliminate the influence of severe weather to the second generation.

In the prediction of the growth period in soybean hybrid progenies, *E1* and *E4* genotypes are major loci considered. We observed that genotypes with *e1-fs* and *e4* alleles are suitable for planting in Mohe or even higher latitude regions, while the genotypes with *e1-nl* or *e1-as* and *e4* alleles were suitable for planting at latitudes from 46°N to 52°N (such as Ganhe, Heihe, and Suihua). The genotypes with the *E4* dominant allele were suitable for planting in the area of latitude 46°N or even in the regions further south. We found that 100% of the super-early group materials could mature in Beijicun Village of Mohe, whereas about 37.2% of the extremely early group materials could mature in Beijicun. We observed that some lines with the same *E1–E4* alleles performed differently in the maturity stage, which might be caused by other genes, such as *FT1a/2a/5a/2b* and *PRR37* (Liu et al., 2017; Cai et al., 2018; Chen et al., 2020; Wang et al., 2020). Future studies should include the effect of these genes on the phenotypes.

To accelerate breeding, numerous methodologies were explored. The use of winter nursery doubled the rate of generation advancement (Gai et al., 2015), while artificial environment with varying photoperiod, temperature, or CO_2_ concentration could achieve three generations in corn (Li et al., 2016); four generations in rice (Tanaka et al., 2016), legumes (Ochatt et al., 2002), and canola (Mobini and Warkentin, 2016; Yao et al., 2016); five generations in soybean (Nagatoshi and Fujita, 2019; Jahne et al., 2020); and six generations in wheat (Kothari et al., 2011) and cabbage (Williams and Hill, 2013). Biotechnologies such as the immature embryo culture or double haploid can shorten one generation time to 65–70 days in soybean (Rosenberg and Rinne, 1987), 50–70 days in cotton (Wang et al., 2003), 39–55 days in barley (Zheng et al., 2013), 66–80 days in sorghum (Rizal et al., 2014), 48–61 days in oat (Liu et al., 2016), and 48–56 days in canola (Tanaka et al., 2016). These approaches with the help of artificial greenhouse were costly and scale limited.

**FIGURE 5:**
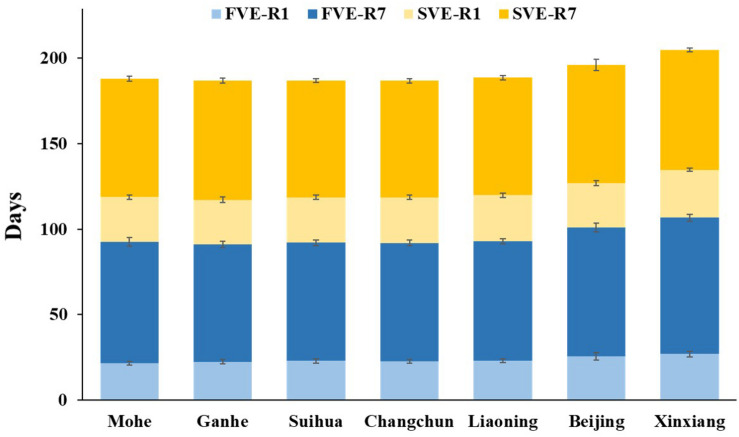
Developmental stages of progenies of cross combinations from Northeastern Spring Planting Region and from Yellow–Huai–Hai Valleys Summer Planting Region planted in Hainan (Winter Nursery). F, first generation; S, subsequent generation; VE, emergence; R1, beginning bloom; R7, beginning maturity.

**FIGURE 6:**
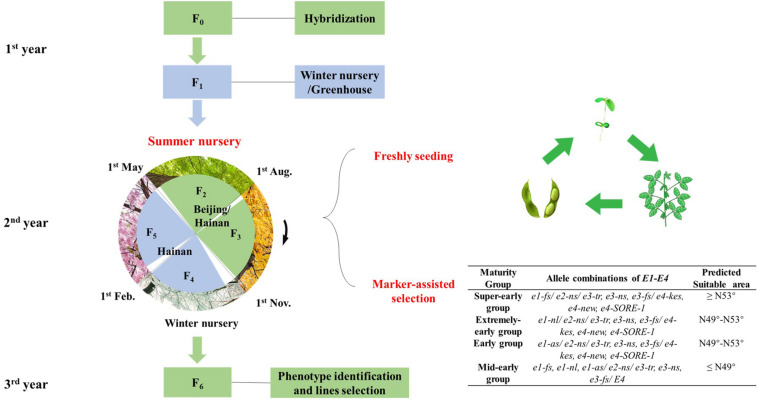
Schematic representation of off-site speed breeding in soybean. The procedure of Speed Breeding System for soybeans from Northeastern Spring Planting Region and Yellow–Huai–Hai Valleys Summer Planting Region to fulfill four generations within 1 year (F_2_–F_5_) and seven generations (F_0_–F_6_) within 3 years.

In this study, the soybean materials from the north part of NE China went through two generations from May to November before the frost in the natural condition of Beijing and were then immediately sent to (November) winter nursery for the next two generations at Nanbin Farm of CAAS in Sanya, Hainan province (Figure 5). We established a speed-breeding system integrating off-site generation advancement and fresh-seeding method, under natural conditions with the accomplishment of at least four or more generations in 1 year (Figure 6). In this study, we summarized the operating procedures as follows:

(1) The hybrid progenies from the north part of NE were planted in the summer of YHH regions such as Beijing, seeded in early May, and the plants were expected to blossom in early June and harvested in late July. Similarly, the hybrid progenies from the middle and south parts of NE and that from YHH could be planted in Sanya, Hainan province in summer, seeded in early May, and the plants were expected to blossom in early June, and harvested in late July.
(2) After the plants reach the full-seed stage (R6), fresh pods with the fastest growth in the lower part of the plants were picked and sowed immediately after separation from pods.
(3) The second generation of hybrid progenies from north part of NE were sown in early August in YHH regions, and harvested in early November. Similarly, the second generation of hybrid progenies from middle and south parts of NE and that from YHH could be sown in early August in Sanya, Hainan province, and harvested in late October.
(4) During the above process, maturity groups and suitable planting area of each individual line could be predicted by identifying *E1–E4* alleles, which could further save the time of phenotypic identification in the target region.

## Data Availability Statement

The original contributions presented in the study are included in the article/Supplementary Material, further inquiries can be directed to the corresponding authors.

## Author Contributions

TH and SS designed and managed the research project. YF, ES, SF, HZ, XS, SQ, MK, and TH conducted the field experiment. YF, TW, LW, and SY conducted the genotype identification. CW and WH provided advice on experimental implementation. YF, TH, LW, and ES took the lead in the manuscript preparation and all authors are qualified for authorship and were involved in drafting and revising this manuscript.

## Conflict of Interest

The authors declare that the research was conducted in the absence of any commercial or financial relationships that could be construed as a potential conflict of interest.

## Publisher’s Note

All claims expressed in this article are solely those of the authors and do not necessarily represent those of their affiliated organizations, or those of the publisher, the editors and the reviewers. Any product that may be evaluated in this article, or claim that may be made by its manufacturer, is not guaranteed or endorsed by the publisher.

## Supplementary Material

Supplementary Figure 1 Day length and ambient temperature in Beijing and Sanya during soybean summer nursery in 2018. The day length data were downloaded from the website https://sunsetsunrisetime.com/sun. The ambient temperature data in Beijing and Sanya were from the website http://lishi.tianqi.com/beijing/index.html and http://lishi.tianqi.com/hainanqu/index.html.

Supplementary Figure 2 Flowering time (days from VE to R1) and maturity time (days from VE to R7) of different *E* allele combinations. Flowering time and maturity time of **(A)** different *E* allele combinations, **(B)** *E1* allele, **(C)** *E3* allele, and **(D)** *E4* allele in Ganhe. VE, emergence; R1, beginning bloom; R7, beginning maturity. One-way ANOVA was used for the statistical analysis (*P* < 0.05).

Supplementary Table 1 The sources and maturity group of parents.

Supplementary Table 2 The primer sequences used in this study.

Supplementary Table 3 The phenotype and haplotype combinations of *E1-E4* in hybrid progenies. “NA” means these materials cannot reach R7 until the first frost came.
