# Supplementary figures and images for: Speed-Breeding System in Soybean: Integrating Off-Site Generation Advancement, Fresh Seeding, and Marker-Assisted Selection
Source: Front Plant Sci. 2021 Aug 17;12:717077. doi: 10.3389/fpls.2021.717077 (PMC8416080; doi:10.3389/fpls.2021.717077)

## Slide 1
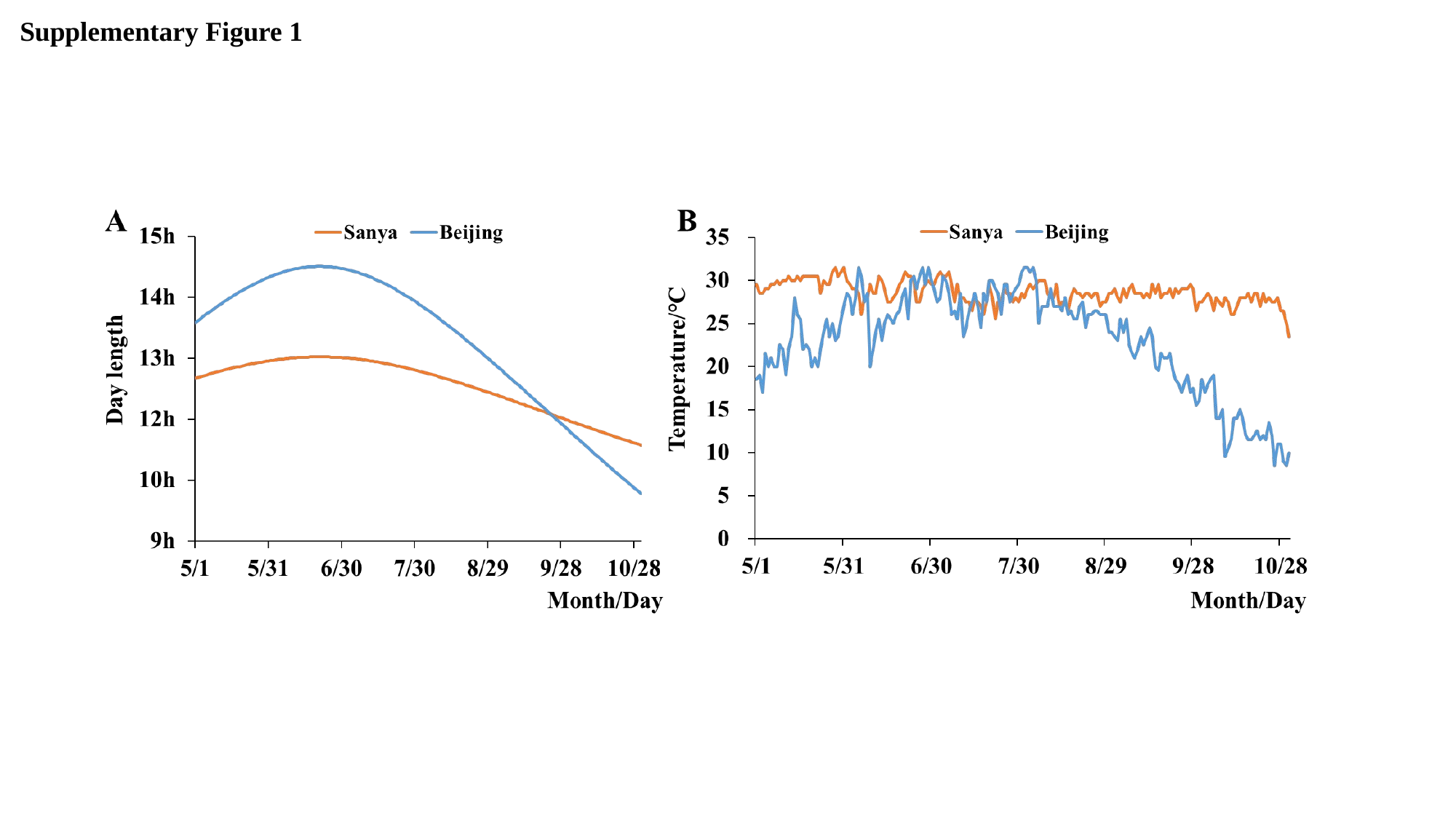

Supplementary Figure 1

## Slide 2
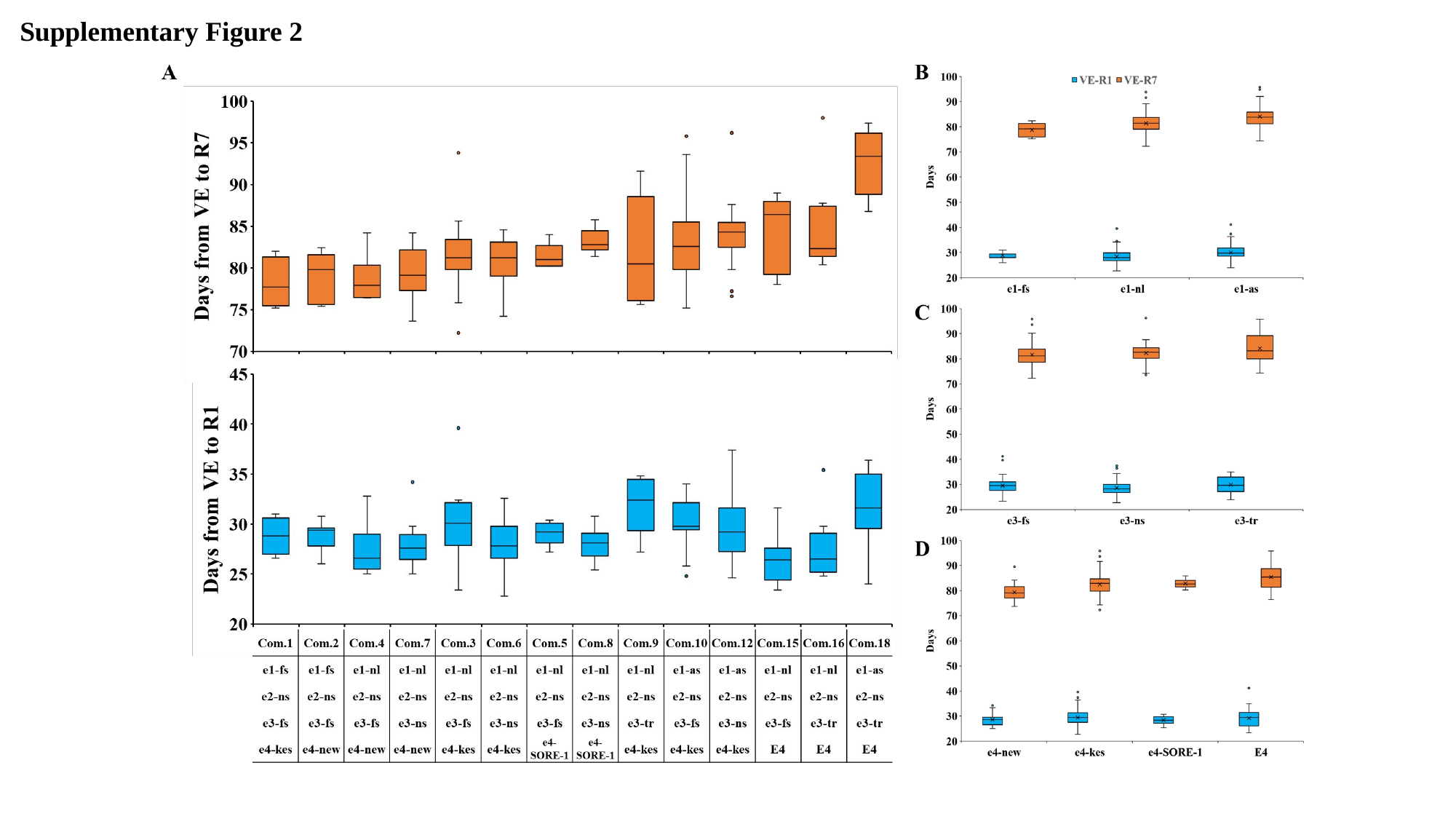

Supplementary Figure 2

Supplement: Supplementary Figure 1 — Day length and ambient temperature in Beijing and Sanya during soybean summer nursery in 2018. The day length data were downloaded from the website https://sunsetsunrisetime.com/sun. The ambient temperature data in Beijing and Sanya were from the website http://lishi.tianqi.com/beijing/index.html and http://lishi.tianqi.com/hainanqu/index.html. [file Presentation_1.PPTX]
